# The ERK 1 and 2 Pathway in the Nervous System: From Basic Aspects to Possible Clinical Applications in Pain and Visceral Dysfunction

**DOI:** 10.2174/157015907782793630

**Published:** 2007-12

**Authors:** Célia D Cruz, Francisco Cruz

**Affiliations:** 1 Institute of Histology and Embryology, Faculty of Medicine and IBMC, University of Porto, Portugal,; 2 Department of Urology, Faculty of Medicine, University of Porto, Portugal

**Keywords:** ERK, MAPK, somatic pain, visceral pain, visceral reflex activity, ERK inhibition, pain.

## Abstract

The extracellular signal-regulated kinases 1 and 2 (ERK) cascade, member of the mitogen-activated protein kinases superfamily of signalling pathways, is one of the best characterized pathways as many protein interactions and phosphorylation events have been systematically studied. Traditionally, ERK are associated with the regulation of proliferation and differentiation as well as survival of various cell types. Their activity is controlled by phosphorylation on specific aminoacidic residues, which is induced by a variety of external cues, including growth-promoting factors.

In the nervous system, ERK phosphorylation is induced by binding of neurotrophins to their specific tyrosine kinase receptors or by neuronal activity leading to glutamate release and binding to its ionotropic and metabotropic receptors. Some studies have provided evidence of its importance in neuroplastic events. In particular, ERK phosphorylation in the spinal cord was shown to be nociceptive-specific and its upregulation, occurring in cases of chronic inflammatory and neuropathic pain, seems to be of the utmost importance to behavioural changes observed in those conditions. In fact, experiments using specific inhibitors of ERK phosphorylation have proved that ERK directly contributes to allodynia and hyperalgesia caused by spinal cord injury or chronic pain. Additionally, spinal ERK phosphorylation regulates the micturition reflex in experimental models of bladder inflammation and chronic spinal cord transection.

In this review we will address the main findings that suggest that ERK might be a future therapeutic target to treat pain and other complications arising from chronic pain or neuronal injury.

## INTRODUCTION

In the past few years, the Mitogen-Activated Protein Kinase (MAPK) family of signalling cascades has received a great deal of attention, especially the ERK 1 and 2 pathway. This signalling cascade is involved in the regulation of a variety of cellular functions, ranging from the government of cell fate and survival to several plastic changes. Its importance in proliferative phenomena has attracted immense attention as it seems to be extremely important for some types of cancers, an issue that outbounds the present review.

In the nervous system, activation the ERK pathway occurs in a variety of locations and situations, some of which contribute to painful conditions. Pain is a complex multi-dimensional experience related to actual or potential tissue damage, according to the International Association for the Study of Pain [72]. It can occur as a symptom of a disease as in the case of chronic inflammation or a disorder in its own right as in case of painful neuropathies. In both cases, several changes occur in the nervous system, which depend on the activation of the ERK pathway at the different neuronal levels, including dorsal root ganglia (DRG), spinal cord and supraspinal centres. This issue will constitute a main topic of the present review.

In viscera, conditions leading to visceral pain are also commonly associated with changes in the reflex control of the affected organs. The events that occur in the neuronal circuitry responsible for the modulation and integration of noxious input arising from viscera and the respective reflex control are still poorly understood. However, there is now strong evidence that the ERK signalling pathway may be extremely relevant. Therefore, this issue will also be thoroughly discussed in this review.

Finally, given the importance of the ERK pathway in a variety of painful disorders and altered visceral reflex control, attention will also be paid to the use of ERK inhibitors to provide analgesia and regulate visceral reflex activity.

## THE MAPK FAMILY OF SIGNALLING PATHWAYS

Eukaryotic cells are constantly submitted to various forms of physical and chemical stress, including contact with neighbouring cells, hormones, growth factors and cytokines. After identification of the external stimulus by membrane receptors, a cellular response programme is set in motion, involving the activation of intracellular signalling pathways composed of sequentially activated enzymes. The final response may be the expression of new genes, modulation of structural elements or alterations in cell cycle progression.

**Fig. (1):**
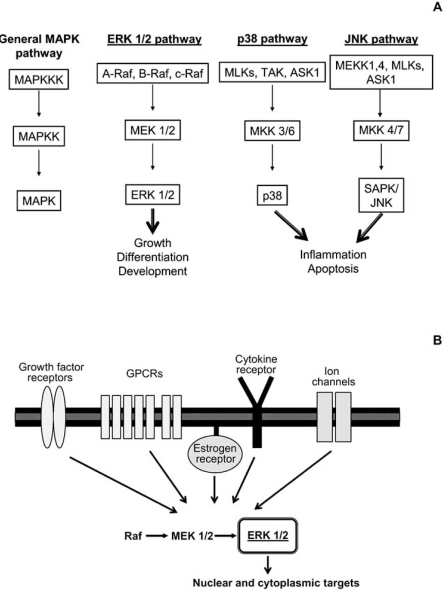
The MAPK family with its classical cascades, the ERK 1 and 2,p38 and JNK. (A) The MAPK family of signalling pathways is characterized by a central motif of three kinases, which activate each other in a sequential order by phosphorylation of specific residues. Classically, 3 distinct pathways are included in the MAPK family (the ERK 1 and 2, p38 and JNK),the activation of which leads to different cellular outcomes. (B) The activation of the ERK pathway depends on a variety of membrane bound receptors,including growth factor receptors, G protein coupled receptors (GPCRs), the estrogen receptor and ionic channels. In some cases, binding of certain cytokines to their specific receptor may also lead to the activation of this pathway. Once active (that is, phosphorylated) ERK can target a variety of nuclear and cytoplasmic elements.

One of the families of signalling pathways that has attracted more attention in the past few years is the MAPK family. As many of the known signalling pathways, such as the Protein Kinase C (PKC) [80], Protein Kinase A (PKA) [75] or the Protein Kinase G (PKG) pathways [107], MAPKs are regulated by phosphorylation, which consists in the covalent binding of phosphate groups to specific aminoacid residues. This process is catalyzed by enzymes designated as kinases [51]. The first members of the MAPK family were identified in the 1980s, when different research groups demonstrated the existence, in several non-neuronal cell types, of a 42 kDa protein, phosphorylated in tyrosine residues upon stimulation with growth factors and phorbol esters [21, 55]. At the same time, two serine/threonine kinases activated by the insulin receptor were characterized [3, 89]. These kinases were named according to their substrate, thus termed myelin basic kinase (MBP) and microtubule-associated kinase 2 (MAP-2). With time, it became evident that these kinases were the 42 kDa protein phosphorylated at the tyrosine residues. They were named MAPK [19] and later Extracellular-signal Regulated Kinase (ERK), to reflect the variety of stimuli that can lead to its activation [8]. Since then, other kinases sharing a high degree of homology with ERK were identified and the term MAPK was used to identify the whole family (Fig. **1**) which includes now 5 distinct branches: ERK 1 and 2; c-Jun N-terminal kinases (JNKs) 1, 2 and 3; p38 (isoforms α, β, γ, δ); ERK 3, 4 and ERK 5; e, more recently ERK 6, 7 and 8 [7]. Interestingly, each branch seems to have a preferential activating stimulus: trophic factors for ERK, UV radiations for p38 and extracellular osmolarity for JNK [14].

Although each MAPK branch has distinctive characteristics, there are common features among the MAPK pathways. The most striking aspect of these signalling cascades is a central core of three serially linked kinases in each pathway (Fig. **1**) and the existence of especial aminoacidic domains in their structure, important for a correct running of MAPK signalling. In what concerns the specificity, duration and magnitude of MAPK signalling, those are assured by several mechanisms that require the participation of structural and accessory proteins. Although important, these aspects of MAPK signalling are not the focus of the present review. More information on these issues can be found elsewhere [47].

### The ERK 1 and 2 Signalling Pathway

The first MAPK to be cloned and properly characterized was ERK 1 [10], quickly followed by ERK 2 [9]. ERK 1 and 2 share an 83% homology in their sequences and are expressed in all tissues [15, 90]. Upstream of ERK, there is the small GTPase Ras, the Ras-activated kinase (Raf) and the MAP kinase kinases (MEK) 1 and 2. Raf, from which 3 isoforms are known, is the first to become active and phosphorylates MEK 1 and 2 at two serine or threonine residues. In turn, MEK phosphorylates ERK at two tyrosine and threonine residues. Dual phosphorylation is crucial for full activation of MEK and ERK [9, 87].

Once active, ERK can phosphorylate various elements located in different sub-cellular compartments. Some of the substrates include membrane proteins (CD120a, Syk and calnexin), nuclear proteins (SRC-1, Pax6, MEF2 and STAT3), cytoskeletal elements (neurofilaments and paxilin) [14, 15, 84, 90] and cytoplasmic kinases, [90], including members of its own pathway [13, 58, 68]. It is this extensive interaction with different types of cellular elements that enable ERK to interfere with a vast array of cellular events [22, 66, 104].

Interruption of ERK signalling may occur by (1) hyperphosphorylation of Raf and MEK 1 and 2, (2) dephosphorylation of aminoacidic residues of ERK and (3) sequestration of ERK in the nucleus. In regard to the levels of phosphorylation of Raf and MEK, it is possible that ERK, apart from phosphorylating downstream substrates, could also phosphorylate upstream targets, such as those two kinases [13, 58, 68] generating a negative feedback dependent on hyperphosphorylation [15, 45].

The removal of phosphate groups depends on specific enzymes known as MAPK phosphatases (MKPs) which dephosphorylate tyrosine, serine/threonine residues or both groups of residues. Although most of these phosphatases are cytosolic, some may be found in the nucleus where they are responsible for ERK sequestration [15, 59, 60, 86, 105, 106]. 

## ERK REGULATES NEURONAL CELL FATE AND PLASTICITY

In the nervous system, the first descriptions of ERK expression and activation date from the early 1990’s and relate to the role of ERK in proliferation and differentiation of PC12 cells, derived from the rat adrenal phaechromocytoma [41]. Nerve growth factor (NGF) triggers cell cycle exit and differentiation of PC12 cells into sympathetic neurons, whereas treatment with epidermal growth factor (EGF) induces PC12 cell proliferation. As part of the response to those trophic factors, a robust activation of the ERK cascade was demonstrated. Interestingly, NGF induced a more long-lasting phosphorylation, with translocation of the active forms into the nucleus and activation of the expression of specific neuronal genes. On the other hand, EGF triggered a brief ERK activation, insufficient to induce nuclear translocation and consequent neuronal differentiation [22, 41, 44, 114].

It is well known that for PC12 cells and neurons, including sympathetic and cerebellar neurons [111] kept in culture, removal of NGF from the culture medium constitutes a strong pro-apoptotic stimulus. However, in these cases, apoptosis can be prevented if cells are transfected with hyper-active forms of kinases from the ERK pathway [69, 112]. Activation of the anti-apoptotic gene *bcl-2* might be the mechanism by which neuronal survival is regulated by the ERK cascade [73].

Although crucial for neuronal survival, one should take in consideration that extremely prolonged activation of the ERK pathway can be deleterious. As a matter of fact, excessively long-lasting neuronal ERK activation has already been demonstrated in neurodegenerative diseases such as Parkinson`s and Alzheimer`s Diseases [32, 46, 57, 91]. 

In what concerns neuronal plasticity, ERK intervene in long-term potentiation (LTP), the basis of the learning process, acquisition and maintenance of long-term memory in mammals [67]. ERK participation in LTP was demonstrated for the first time in 1996. *In vitro* studies showed the occurrence of ERK activation in the CA1 area of the hippocampus after stimulation of the glutamate N-methyl-D-aspartate (NMDA) ionotropic receptor [33, 34]. In latter studies it was further demonstrated that ERK also participate in NMDA-independent forms of LTP [20] and their activation can also occur in other areas of the hippocampus, including the dentate gyrus. Today it has been accepted that, besides the hippocampus, ERK also play a part in LTP occurring in the amygdala, insula and in the synapses between thalamic and amygdalar neurons [52, 93]. In a general way, ERK activation seems to contribute to LTP by controlling the expression of genes such as *zif268* and a*rc* [88, 113]. 

Given that ERK 1 and 2 intracellular signalling pathway plays an important role in the acquisition and consolidation of long-term memory, it would be reasonable to expect that suppression of one of the genes that code for elements of this cascade would alter those functions. Thus, it was shown that knockdown of a Ras accessory protein (involved in the initial steps of activation of this signalling pathway) affects the normal function of the amygdala and the consolidation of long-term memory [11]. Nonetheless, surprisingly, knockdown of the gene coding for ERK1 did not alter the establishment and consolidation of long-term memory [70, 99], although no satisfying explanation for this has been forwarded.

## ERK ACTIVATION BY ACUTE NOXIOUS STIMULI

The first indication of ERK involvement in the processing of noxious stimuli was provided in 1999 by Ji and collaborators. They demonstrated the occurrence of ERK phosphorylation in cells located in laminae I and IIo following electrical stimulation of nociceptive afferents or peripheral stimulation with capsaicin. Spinal ERK phosphorylation was ipsilateral to stimulation, NMDA-depen-dent and necessary for pain behaviour during the 2^nd^ phase of the formalin test. Based on these results, it was proposed that spinal ERK phosphorylation was involved in the generation of pain hypersensitivity [48].

Subsequent studies further confirmed ERK activation occurred in response to acute noxious stimulation of primary afferent neurons, [37, 53, 54], the predominant location being the superficial laminae of the ipsilateral dorsal horn [53, 54]. ERK phosphorylation was strictly restricted to neurons [24, 27] and short-lived, basal levels being reached 1 to 2 hours after stimulation [37, 48, 53]. Similarly to what had been observed in the spinal cord, ERK activation was also observed in the trigeminal nucleus, following perioral injection of formalin [43].

**Fig. (2):**
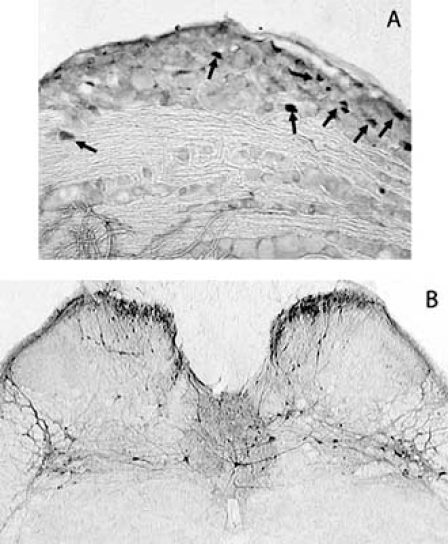
PhosphoERK immunoreactive neurons in sections from the L6 dorsal root ganglion (A) and spinal cord (B). Animals were submitted to acute noxious bladder distension. ERK activation occurred mostly in smallto medium diameter neurons (A, arrows), most likely nociceptive neurons.In the spinal cord (B), immunoreactive neurons were located bilaterally in the superficial laminae of the cord, in the dorsal commissure and in the intermediolateral grey matter, areas known to receive bladder sensory input (Cruz *et al*., unpublished observations).

In the DRG of non-stimulated animals, levels of ERK activation are very low and mostly restricted to small diameter cells, presumably nociceptive neurons. ERK phosphorylation in response to acute noxious stimulation has also been described in these cells. ERK activation was observed in the perycaria of small primary afferent neurons [29, 30, 83, 94] (Fig. **2**) but it also occurred in the peripheral processes of these cells [4, 6, 29, 30]. Curiously, phosphoERK levels were found increased in primary afferents following exposure to NGF and seem to be important for the retrograde transport of that neurotrophin [6].

In supraspinal nuclei, activation of the ERK pathway in response to acute noxious stimulation is still poorly documented. The few studies available refer the occurrence of immunoreactive cells for the phosphorylated forms of ERK in the brainstem and in several structures of the tele- and diencephalon following noxious stimulation, including the hypothalamus, the paraventricular nucleus of the thalamus, the parabrachial nucleus, the dorsal raphe nucleus [16, 38, 39, 40]. ERK activation has also been shown to occur in the hippocampus (dentate gyrus and CA3 zone) and hypothalamic paraventricular nucleus following intrathecal injection of Substance P [16]. 

## ERK ACTIVATION BY CHRONIC NOXIOUS STIMULI

ERK activation by chronic noxious stimulation is not particularly different from that seen after acute noxious stimulation. In the vast majority of situation, ERK activation occurs in the same areas. The differences rest on the more intense levels of ERK phosphorylation and longer duration [2, 6, 24, 27, 36, 49, 83, 96]. In addition, as an additional distinction from acute noxious stimulation, in some models of neuropathic pain ERK activation was also found in non-neuronal cells (see below).

In animals with chronic inflammation of the hindpaw or joint, spinal ERK activation was upregulated and became persistent, with levels remaining elevated up to a maximum of 3 days [27, 49]. Furthermore, in the case of chronic joint inflammation, ERK phosphorylation was not only upregulated but also occurred in deep laminae of the cord [27], probably reflecting changes occurring in neuronal circuitry associated with chronic joint pain [79, 92]. 

In what concerns spinal ERK phosphorylation after visceral inflammation, the scarce studies available show that noxious stimulation of the chronic inflamed colon and bladder induces prolonged and intense ERK activation in the lumbosacral spinal cord [24, 36] where most of the primary afferents innervating these organs terminate. Thus, in the case of chronic cystitis, ERK phosphorylation occurred bilaterally in neurons located in the superficial dorsal horns, intermediolateral grey matter areas and dorsal commissure [24] (Fig. **2**). 

Spinal ERK phosphorylation has also been addressed in models of neuropathic pain. In this case, ERK phosphorylation was found in the cytoplasm and nuclei of spinal neurons [31, 62, 102, 117] but also in glial cells, including microglia and astrocytes [31, 63, 117]. Curiously, in animals with spinal nerve ligation, ERK phosphorylation followed a specific pattern. It was firstly observed in neurons, then in microglia (between days 1 and 3 after surgery), in astrocytes and microglia (day 10) and, finally, appeared restricted to astrocytes (day 21) [117]. In the DRG, in contrast with acute noxious stimulation or inflammation, ERK phosphorylation occurred in medium-to-large neurons [81, 82, 83], as well as in satellite cells [82, 83], reflecting the involvement of A-fibres and glial cells in neuropathic pain.

## WHY NOXIOUS STIMULI LEAD TO ERK ACTIVATION

The reasons why ERK phosphorylation is upregulated by acute and chronic noxious conditions are various but a common denominator can be found. In all cases studied increases in the spinal levels of excitatory aminoacids and neurotrophins have been reported. It is widely accepted that during inflammation peripheral levels of neurotrophins, ATP, protons, bradykinin among others, are upregulated, leading to sensitization of peripheral sensory fibres [109, 110]. Thus, they are more likely activated by stimulation and will release increased amounts of neurotrophins, retrogradely transported from peripheral tissue or produced in the soma [56], and glutamate in the spinal cord. As the main focus of the present review is not pain, further detail on this subject can be found elsewhere [74].

**Fig. (3):**
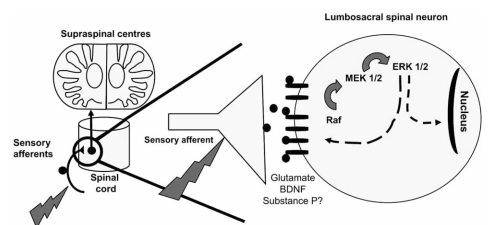
Mechanisms of ERK activation in the spinal cord. Upon noxious peripheral stimulation, glutamate and BDNF are released onto the spinal cord. Upon binding to their respective receptors, activation of the pathway occurs in the cytoplasm of spinal neurons. Once activated, ERK can modulate the activity of membrane receptors by phosphorylating specific subunits. Activated ERK can also translocate to the nucleus and induce gene transcription by phosphorylation of transcription factors. The importance of substance P is still in debate.

The identification of membrane receptors that lead to downstream ERK phosphorylation has been widely addressed. Available data points to the involvement in the spinal cord of both ionotropic [48, 54, 61], or metabotropic 1 and 5 glutamate receptors [53, 54, 61, 96]. Binding of brain-derived neurotrophic factor (BDNF) to its specific TrkB receptor is also important for spinal activation of the ERK pathway [85, 100] (Fig. **3**). The importance of the spinal substance P (SP) receptor (the NK1 receptor) is, at present less clear. In fact, while some studies demonstrated that SP induces spinal ERK activation [54, 108], other have provided opposing data [61]. Finally, the contribution of other signalling pathways should not be ruled out as it has been verified that activation of the PKC and PKA pathways may lead to ERK phosphorylation [54, 108].

Traditionally, spinal ERK phosphorylation has been assumed to depend exclusively on sensory afferent input. Current data, however, shows that supraspinal serotonergic input may also contribute to ERK activation in the spinal cord. Spinal 5-HT3 receptors seem to be involved as intrathecal administration of a selective antagonist of this receptor attenuates ERK activation induced by formalin injection in the paw [103]. On the other hand, supraspinal input may also inhibit spinal ERK phosphorylation. As such, in animals with chronic spinal cord transection ERK phosphorylation was shown to be upregulated [see below; 26]. However, the nature of this inhibitory supraspinal input is still to be identified.

In the peripheral nervous system, the ATP receptor, P2X3, seems to play an important role in regulating ERK phosphorylation in DRG neurons, especially in models of inflammatory joint pain [6, 83]. Lastly, inter-connections with other signalling pathways in DRG neurons, namely the phosphatidylinositol 3-kinase (PI3K) pathway [118], appear to also conduct to ERK phosphorylation.

In what concerns ERK phosphorylation increase in glia cells in cases of neuropathic pain, a growing body of evidence indicates they play a pivotal role in this form of chronic pain [for review see 71]. ERK activation might be important for the expression of pro-nociceptive enzymes and cytokines including inducible nitric oxide synthase (iNOS), cyclooxygenase-2 (COX-2), interleukin-1beta (IL-1β), tumour necrosis factor alpha (TNF-α) and interleukin-6 (IL-6), making the ERK pathway a key signalling pathway in glia cells. Nonetheless, there are currently no direct data supporting these deductions. Further studies are necessary to clarify the significance of ERK phosphorylation in glia cells as well as to define the precise stimuli that produce ERK activation in this type of cells. 

## ERK ACTIVATION FOLLOWING SPINAL CORD INJURY

Very few studies have concentrated on the effects of spinal cord lesions on the levels of ERK phosphorylation. Nevertheless, available data indicates that spinal injuries such as contusion, excitotoxic injury or chronic complete transection lead to upregulation of spinal levels of ERK phosphorylation [23, 26, 115]. In the cases of contusion and excitotoxicity, ERK phosphorylation was particularly upregulated in the proximal areas (caudal and rostral in relation to the injury site) [23, 115]. Furthermore, these high levels of ERK activation were correlated with increased expression of the receptor NK1, NMDA subunits NR1, NR2A [115] and the transcription factor cAMP response element binding protein (CREB) [23]. Such changes were only observed in animals that displayed injury-induced pain behaviour.

Interestingly, in cases of chronic spinal cord transection at high thoracic segments, ERK phosphorylation was upregulated in L6 segment, that is, in a distal segment to the injury site. This is the segment that receives the majority of bladder-generated sensory input [76, 77]. In cases of spinal cord injury such sensory input is upregulated and its source switched from Aδ to C-fibres [28].

In models of cord injury, as in pain models, ERK phosphorylation seems to rely on excitatory aminoacids and neurotrophins. In fact, it has been well documented that spinal cord lesions produce excessive glutamate release at the lesion site and in its vicinity [78]. Furthermore, in paradigms of spinal injury-induced bladder hyperactivity, the importance of neurothrophins is quite established with high amounts of BDNF and NGF being observed in both the urinary bladder and spinal cord in animals with chronic spinal transection [97, 98, 119]. 

## CONSEQUENCES OF ERK ACTIVATION

The reasons why ERK activation in the nociceptive system may contribute to altered pain states is presently under active investigation. So far, it seems clear that ERK contribution is related to control of gene expression and interaction with membrane receptors. These events may occur in the peripheral and central nervous system either independently or in a concurrent fashion.

In the DRG, ERK activation has been shown to be responsible for the upregulation of BDNF levels in the cell bodies of DRG neurons in models of peripheral inflammation and neuropathic pain [81, 82, 83]. Furthermore, in a model of neuropathic pain induced by chronic constriction of the sciatic nerve, ERK phosphorylation in the DRG was shown to lead to increases in the levels of neuropeptide Y (NPY) in damaged neurons [81]. In all cases, blockade of ERK phosphorylation lead to downregulation of the contents of BDNF and NPY, correlating with improvement in pain hypersensitivity. 

ERK activation in DRG neurons may also contribute to altered heat sensitivity by interacting with the transient receptor potential vanilloid receptor-1 (TRPV1), as demonstrated by Zhuang *et al*. (2004) [118]. Accordingly, inhibition of ERK activation almost completely inhibited the facilitation of heat-induced currents in DRG neurons [35]. Furthermore, ERK inhibition has been shown to partially reduce the upregulation of TRPV1 expression induced by NGF. However, in this process the participation of other Ras-dependent signalling pathways should also be taken into consideration [12].

Finally, ERK phosphorylation may also be accounted to participate in the development and establishment of opioid tolerance which is thought to derive, at least in part, from increased activity of nociceptive pathways. In fact, *in vitro* studies have shown upregulation in the levels of substance P and calcitonin gene related peptide (CGRP) in DRG neurons chronically exposed to morphine [64]. Such upregulation was shown to be mediated by ERK [65]. 

In the spinal cord, ERK phosphorylation is important to modulate the activity of the NMDA receptor and the potassium channel Kv 4.2. Upon noxious stimulation and subsequent release of BDNF onto the spinal cord, activated ERK phosphorylates the NR1 subunit of the NMDA receptor [101]. This phosphorylation increases its opening probability, leading to increased neuronal excitability. In what concerns the Kv 4.2 channel, recent studies demonstrate that ERK can phosphorylate the pore-forming subunit of this channel and contribute to pain plasticity at the spinal cord level [1, 42].

Regarding the contribution to the regulation of neuronal gene expression, ERK phosphorylation was shown to be crucial for the upregulation of the spinal levels of NK1 and prodynorphin seen in animals with hindpaw inflammation [49]. Another spinal gene whose expression is believed to be regulated by ERK is c-*fos*, a pain-evoked immediate early gene, in spite of the evidence that in non-spinal neuronal cells c-*fos* expression may occur without ERK participation [50]. In the spinal cord neurons, upon ERK blockade with specific inhibitors delivered intrathecally, a reduction was observed in spinal *c-fos* expression induced by noxious somatic [54] and visceral stimulation [25]. 

## BLOCKING ERK: A NEW THERAPEUTIC TARGET?

**Fig. (4):**
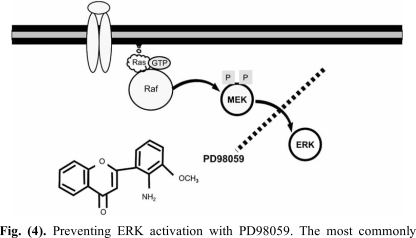
Preventing ERK activation with PD98059. The most commonly used approach to avert ERK phosphorylation consists in blocking the interaction between phosphorylated MEK and inactive ERK. Several inhibitors have been developed to accomplish this task, the most frequently used of which is PD98059. This inhibitor is a non-competitive cyclic molecule with an amino moiety.

Because in most studies ERK phosphorylation was found in neurons involved in nociception at both the peripheral and central nervous system and upregulated by chronic inflammation, it was hypothesized that it should constitute an important mechanism for pain perception. Furthermore, because high levels of ERK activation correlated with the occurrence of both allodynia and hyperalgesia in several pain models, it is likely that this mechanism should contribute to plastic neuronal changes associated with chronic pain. On this ground, inhibitors of ERK phosphorylation have been used in order to reverse those altered pain states. The most commonly used strategy to prevent ERK activation is by blocking the activation of ERK by its upstream kinase MEK (Fig. **4**). The classical molecules used to fulfil this purpose were the MEK inhibitors U0126 and PD98059. This last compound proved to be useful to reduce the second phase of the formalin test when given as an intrathecal infusion [17, 48]. Although several routes of administration have been tested, including intravenous [36], intradermal [30], joint [95], intrathecal [24, 27, 48] and intracerebroventricular injections [17], they should be used with care as the site in which ERK inactivation occurs may be difficult to ascertain **(**such as in the case of intravenous injection**)** and the role of ERK phosphorylation in peripheral fibres is still poorly understood.

**Fig. (5):**
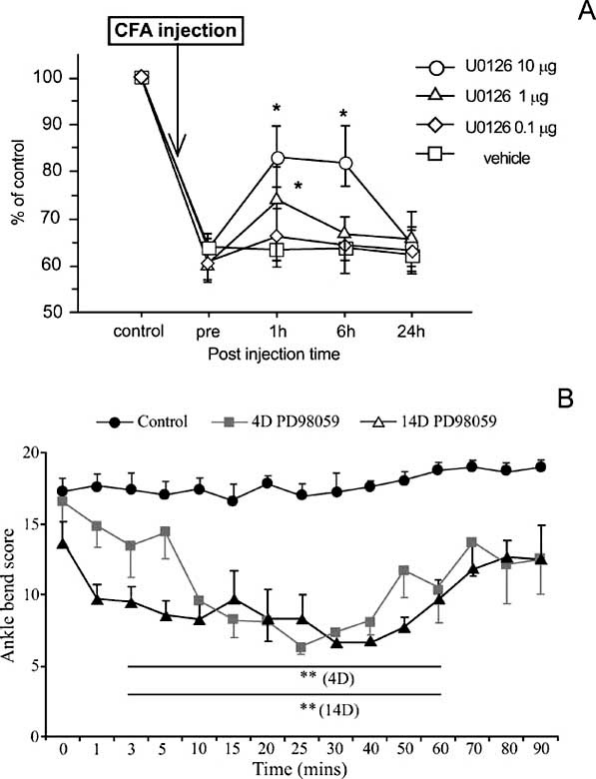
Reduction of allodynia in animals with chronic joint inflammation following administration of ERK inhibitor. In (A), U0126 (another ERK inhibitor) was delivered an intra-articular injection. As a consequence, the angle of knee movement, which was less than 70% of control, was improved with the administration of 1 and 10µg of intra-articular inhibitor. Adapted from [95]. In (B), ankle-bend scores for saline- and intrathecal injected PD98059 in monoarthritic (MA) rats at different time points. The anklebend test for MA rats was performed immediately before the intrathecal injection (time 0) of either saline (control; black circles) or 1 µg (grey squares) and 2 µg (white triangles) of PD98059 in MA rats with 4 and 14 days of evolution, respectively. The high struggle scores induced by ankle bending observed in control MA animals was increased thought-out the all experimental period. On the contrary, ankle-bend scores for the PD98059 injected groups were significantly decreased. Adapted from [27]. CD Cruz,Pain (2005) 116:411-419. Used with permission. D Seino, Pain (2006)123:193-203. Used with permission.

Nevertheless, available results show that intrathecal administration of U0126 or PD98059 successfully attenuated mechanical allodynia in cases of inflammatory [17, 36, 54, 82, 83, 96] and neuropathic pain [81, 82, 102, 117], heat hypersensitivity [2, 5, 17, 29, 30] and mechanical hyperalgesia [30, 31]. In the particular case of chronic hindpaw inflammation, ERK blockade also prevented the upregulation in the spinal levels of prodynorphin and NK1 receptor observed in such animals [49]. Furthermore, ERK inactivation also lead to reduction of neuropathic static allodynia [18], suppression of autotomy [83], decreased referred hyperalgesia following colon inflammation [36] and reduction in nociceptive behaviour derived from joint inflammation [27, 96]. Interestingly, it should be noted that the use of ERK inhibitors in animals with chronic joint inflammation to minimise allodynia allowed the evaluation of the effects of ERK activation at different sites. Intra-articular administration of U0126 in animals with knee inflammation clearly improved the struggle threshold (Fig. **5A**), [96]. This serves as evidence of the importance of ERK phosphorylation in the peripheral processes of sensory neurons. Also in animals with chronic joint inflammation, intrathecal administration has been tested and a reduction in the levels of allodynia was also found (Fig. **5B**), [27]. This indicates that ERK phosphorylation in spinal neurons or at the central processes of joint primary afferents is vital for the decreased mechanical threshold observed in those animals.

**Fig. (6):**
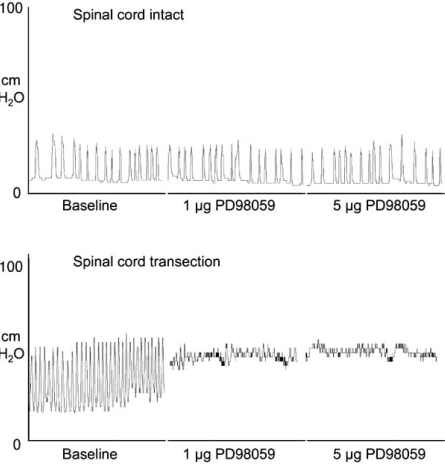
Effect of intrathecal administration of PD98059 on bladder reflex activity in animals with intact and chronically transected spinal cord. In animals with intact cords, PD98059 had no effect in bladder contractions,despite the amount of inhibitor injected. In animals that underwent chronic spinal cord transection at high thoracic levels, bladder reflex activity is clearly altered. These animals display high frequency of bladder contractions with strong micturition pressures. PD98059 visibly reduced both the frequency and amplitude of bladder contractions (Cruz *et al*., unpublished results).

Additionally, available data also showed correlation between high levels of ERK phosphorylation in lumbosacral spinal cord and bladder hyperactivity caused by chronic bladder inflammation and spinal cord transection. In both cases, intrathecal injection of PD98059 strongly reduced the frequency [24, 26] and amplitude of bladder contractions [26] (Fig. **6**), indicating that spinal ERK phosphorylation is important to regulate micturition in pathological conditions. Also relevant to the eventual therapeutic application of ERK inhibitors was the finding that administration of the same doses of PD98059 in intact animals did not produce any effect whatsoever on bladder reflex activity, including the frequency and amplitude of bladder contractions [24, 26]. This finding may indicate that ERK inhibition might prove to be a good therapeutic strategy, at least for the treatment of bladder dysfunction. 

## CONCLUDING REMARKS AND FUTURE PERSPECTIVES

From the present review it becomes clear that the activation of the ERK signalling pathway might be a key mechanism in the development and maintenance of altered pain states. ERK activation occurs in both glia cells and neurons located in the peripheral and central nervous system, and is strongly upregulated in conditions associated with increased sensory input. Phosphorylated ERK can exert its effects by controlling gene regulation and/or modulating membrane receptors and ionic channels. By preventing ERK phosphorylation, the levels of allodynia and hyperalgesia are reduced. Such results clearly indicate that this signalling pathway might constitute an attractive target for pain relief. As seen above, the classical inhibitors PD98059 and U0126 have been used in several models of pain. Intense efforts have, however, been made in order to develop other inhibitors with increased efficiency, specificity and less prone to cause side-effects [for review see 95]. Studies have focused in 3 proteins: Ras, Raf and MEK. Different types of inhibitors have been developed, ranging from modified nucleotides to small-molecule inhibitors. Interestingly, some of these inhibitors may be administered orally and are already available in the market. Nevertheless, in the available literature, no data was found regarding the use of these new inhibitors to treat pain, a gap which hopefully will be corrected in a near future. 

Furthermore, the activation of this cascade seems also to be highly important in the regulation of visceral reflex activity in animal models of chronic cystitis and spinal cord transection. In patients with conditions mimicked by these models, bladder reflex activity is hugely increased, with patients often complaining about intense urinary frequency and incontinence. The ability of ERK inhibitors to decrease the frequency and amplitude of bladder contractions in inflamed or cord transected animals without affecting bladder reflex activity in intact animals forwards an exceptional therapeutic window for this type of drugs, which should be actively pursued in the very next future. 

Finally, when considering using ERK inhibitors as therapeutic tools, it should be taken into consideration the ubiquitous and vital role played by this pathway for cell survival. Adjusting the characteristics and dosage of ERK inhibitors will be of capital importance. Also, given the organization of the ERK pathway and the interaction with other signalling cascades, the development of new and more powerful inhibitors will necessary aim to target protein-protein interactions, rather than just interfering with enzymatic activity. Although such goal seems difficult to achieve, overcoming this problem may have enormous impact in pain therapeutics and management of bladder dysfunction.

## ABBREVIATIONS

BDNF: = Brain derived neurotrophic factor
CGRP: = Calcitonin gene related peptide
COX-2: = Cyclooxygenase-2
CREB: = cAMP response element binding protein
DRG: = Dorsal root ganglia
EGF: = Epidermal growth factor
ERK: = Extracelular signal regulated kinase
IL-1β: = Interleukin-1beta
IL-6: = Interleukin-6
iNOS: = Inducible nitric oxide synthase
JNKs: = Jun kinases
LTP: = Long term potentiation
MAP-2: = Microtubule associated kinase 2
MAPK: = Mitogen activated protein kinase
MBP: = Mielin basic protein kinase
MEK: = Mitogen-activated protein kinase kinase
MKPs: = MAPK phosphatases
NGF: = Nerve growth factor
NK1: = Neurokinin 1
NMDA: = N-methyl-D-aspartate
NPY: = Neuropeptide Y
PD98059: = [2-(2’-amino-3’-methoxyphenyl)-oxanaphthalen-4-one]
PKA: = Protein kinase A
PKC: = Protein kinase C
PKG: = Protein kinase G
Raf: = Ras effector
SP: = Substance P
TNF-α: = Tumour necrosis factor alpha
TRPV1: = Transient receptor potential vanilloid receptor-1
U0126: = 1,4-diamino-2,3-dicyano-1,4-bis[2-aminophenlythio]butadiene
